# Use and effectiveness of Pioneer re-entry device for subintimal true lumen re-entry: single-centre data and a review of the literature

**DOI:** 10.1186/s42155-021-00268-w

**Published:** 2021-12-02

**Authors:** Yvonne Tsitsiou, Jadesola Ekpe, Laura Harris, Elika Kashef, Mohamad Hamady

**Affiliations:** 1 Department of Interventional Radiology, St Mary’s Hospital, Imperial College Healthcare NHS Trust, London, UK; 2 Department of Surgery and Cancer, Imperial College, London, UK

**Keywords:** Subintimal angioplasty, Re-entry device, IVUS, Recanalization, Pioneer

## Abstract

**Introduction:**

During subintimal angioplasty (SIA), it is not always possible to re-enter the vessel lumen due to a variety of factors. Recanalization using hydrophilic wires and catheters alone, apart from its potential technical failure, is also limited by minimal control over the re-entry point. This is frequently well beyond the point of occlusion, thus often compromising important collaterals. In order to bypass the obstruction and attain controlled re-entry into the lumen of the diseased vessel, a re-entry device (RED) may be required. This paper assesses our centre’s experience with the safety and efficacy of the Pioneer re-entry system and systematically reviews the pertinent literature.

**Method:**

A single centre retrospective study of subintimal angioplasty involving the use of the Pioneer Plus intravascular guided reentry catheter was performed. Patient demographics including age, gender, risk factors, comorbidities clinical indication and complications were recorded. Lesion characteristics, including location and severity of calcification were also assessed.

A systematic literature review of all reported studies where the Pioneer RED was used for iliac and lower limb revascularization was conducted by 2 of the authors using the PubMed (MEDLINE) and EMBASE databases.

**Results:**

The study comprised 30 cases. Technical success was 97%. A small, quickly resolved extravasation was the only device related complication. These results are in line with the systematic review which identified 16 studies using the Pioneer RED, reporting a technical success rate of 87.4–100% (median = 100%) and complication rate of 0–25.8% (median = 0%). However, due to heterogeneity in definitions of technical success, data was not pooled.

## Introduction

Peripheral arterial disease (PAD) is common, with a prevalence of 15–20% in people over 70 years old (Selvin & Erlinger, 2004; Criqui et al., 1985; Hiatt et al., 1995). Patients with intermittent claudication or critical limb ischaemia (CLI) may require revascularisation, achieved via angioplasty, stenting or bypass surgery (Peach et al., 2012; Adam et al., 2005).

Percutaneous revascularisation of chronic total occlusions (CTO) is frequently achieved by passing hydrophilic wires and catheters in the subintimal plane of the occluded vessel. Technical success for subintimal angioplasty (SIA) is 85.7% (Bown et al., 2009), which is limited primarily by failure of re-entry to the true lumen, inaccuracy of re-entry and increasing risk of complications (Lipsitz et al., 2003; Jacobs et al., 2006). In order to bypass the obstruction and re-enter the lumen of the diseased vessel, a re-entry device (RED), such as Intra-Vascular Ultra Sound Pioneer® (Philips, San Diego, California) and fluoroscopically guided Outback® (Cordis, Bridgewater, New Jersey), may be required (Kokkinidis et al., 2020).

The Pioneer device is a 6 French compatible dual lumen monorail catheter tracking over a 0.014-in. wire. A 20 MHz intravascular ultrasound (IVUS) transducer is at the tip, designed to facilitate wire re-entry by allowing visualisation of vessel morphology and aiding in identifying blood flow. The catheter also has a curved retractable needle tip, which is projected from the IVUS catheter at the 12 o’clock position. The true lumen location is verified with IVUS by colour-flow mode and allows penetration of the intimal membrane with a gradual needle depth of up to 9 mm and facilitates subsequent advancement into the true lumen (Rezq et al., 2013).

To date, there are limited published studies about the Pioneer device. This single centre experience study assesses the safety and efficacy of the Pioneer RED together with a systematic review of the existing literature pertinent to this device.

## Method

Ethical approval for this retrospective study was waived according to institutional guidelines. Consecutive patients with vascular disease from a single centre who underwent peripheral intervention from 2015 to 2021 involving the use of the Pioneer re-entry device were included.

Demographic data including age, sex, ethnicity and risk factors such as diabetes mellitus, hypertension smoking and dyslipidaemia were recorded along with clinical presentation, Rutherford classification and lesion characterisation. Severity of calcification was graded using the modified peripheral arterial calcium scoring system (Rocha-Singh et al., 2014). Calcification grading is summarised in Table 1. Technical success was defined as re-entry distance of less than 1 cm from the optimal angiographically-defined target vessel, based on Krishnamurthy and colleagues definition (Krishnamurthy et al., 2010). Clinical success was defined as prevention of amputation.

**Table 1:** Disease severity of patient cohort

Disease Severity	Number of patients
**Rutherford classification**	
**1**	**0**
**2**	**0**
**3**	**7**
**4**	**8**
**5**	**11**
**6**	**4**
Peripheral Arterial Calcium Score^*^	
**1a**	**5**
**b**	**3**
**c**	**2**
**2**	**0**
**3a**	
**b**	
**c**	**1**
**4a**	
**b**	**5**
**c**	**14**

^*^ Modified PACSS. Grade 0; no calcification. Grade I; < 5 cm, Grade II; ≥5 cm length, Grade III (bilateral); < 5 cm length, Grade IV (bilateral); ≥5 cm

a; intimal (linear calcification near the lumen on one side of the vessel), b; medial (calcification in the wall away from the lumen, on one side), c: mixed on one side of the lumen

Statistical and data analysis were performed using Microsoft Excel.

### Systematic review and evidence synthesis

A systematic review of the literature was performed following the PRISMA (Preferred Reporting Items for Systematic reviews and Meta-Analyses) selection process (Moher et al., 2009). PubMed (MEDLINE) and EMBASE were searched by two authors (YT and JE) independently to identify all clinical studies reporting the use and efficacy of the Pioneer re entry device in patients with peripheral arterial disease undergoing subintimal angioplasty of occluded iliac, femoral and popliteal arteries. The literature search included the key terms ‘pioneer’/‘IVUS’ and ‘re-entry’/‘recanalisation’ and ‘peripheral vascular disease’ and alternatives. Our literature search was completed on the 20th February 2021 and included both full-text articles, conference abstracts of cohort and randomised control trials from 2000 onwards.

Inclusion criteria were articles in English with a minimum of 3 cases reporting the use of the Pioneer device for true-lumen RED.

Exclusion criteria included review articles, letters to the editor, editorial reports, case reports, duplicate publications and animal studies. Studies on coronary arteries and aortic dissection were also excluded. The data extracted from the selected studies included publication details, study design, number of patients and their demographics as well as outcome measures such as technical success, any reported measurements of re-entry accuracy and periprocedural complications. Data was tabulated and presented numerically (Table 2) however data was not pooled due to heterogeneity of definitions of technical success and complications.

**Table 2:** Results from studies assessing the use and safety of IVUS re-entry devices. *Result includes data for outback re-entry device ** Conference abstract (Krishnamurthy et al., 2010; Baker et al., 2015; Al-Ameri et al., 2009; Scheinert et al., 2005; Vuruskan & Saracoglu, 2017; Zambrano et al., 2014; Smith et al., 2011; Saketkhoo et al., 2004; Arslan et al., 2014; Saket et al., 2004; Kickuth et al., 2006; Massimi et al., 2013; Gandini et al., 2015)

Systematic literature review data					
Study	No of Cases	Success (%)	Age (years)	Location of CTO	Complications (%)
Saketkhoo et al. 2004 (Rocha-Singh et al., 2014)	6	6 (100)	64–85	2 SFA, 4 CIA	0 (0)
Saket et al. 2004 (Krishnamurthy et al., 2010)	7	7 (100)	73.4	3 FPA, 4 iliac	0 (0)
Scheinert D et al. 2005 (Moher et al., 2009) **	25	25 (100)	63	SFA	0 (0)
Jacobs et al. 2006 (Saketkhoo et al., 2004)	21 (20 patients)	21 (100)	61*	18 iliac, 3 SFA	4 (18)
Kickuth R et al 2006 [German] (Baker et al., 2015)	12 (5 CTOs, 7 aortic dissection)	12 (100)	64.6 ± 12.0	2 CIA, 3 SFA	0 (0)
Al-Ameri et al. 2009 (Arslan et al., 2014)	21	20 (95)	n/a	CIA, SFA	1 (5)
Krishnamurphy et al. 2010 (Vuruskan & Saracoglu, 2017)	11	11 (100)	69.8 ± 2.1	Iliac - 7 CIA, 1 EIA, 3 CIA and EIA	n/a
Smith et al. 2011 (Saket et al., 2004)	8	8 (100)	58.5	SFA	0 (0)
Massimi et al. 2013 (Scheinert et al., 2005) **	36 (72 RED)	69 (96)	n/a	Iliac	n/a
Zambrano et al. 2014 (Kickuth et al., 2006) **	42 (36 patients)	39 (93)	65 (46–86)	5 CIA, 1 EIA, 33 SFA, 3 IPA	2 (5)
Gandini et al. 2015 (Al-Ameri et al., 2009) **	20	18 (90)	n/a	SFA	0 (0)
Baker et al. 2015 (Smith et al., 2011)	20	18 (90)	69 ± 13 years	10 CIA, 3 EIA, 5 SFA	0 (0)
Arslan et al. 2015 (Massimi et al., 2013) **	7	7 (100) *	n/a	lower extremities	0 (0)*
Vuruskan et al. 2016 (Zambrano et al., 2014)	31	30 (96.7)	62.1 ± 9.2*	CIA, EIA, SFA, popliteal	8 (25.8)*
Kokkinidis et al. 2019 (Kokkinidis et al., 2020)	28	(90) *	63.9 (10.9)*	CIA	2 (5)*
Sheikh et al. (2021) (Sheikh et al., 2021)	135	118 (87.4)	70.1 ± 9.4	Iliac and femoropopliteal	(9.6)

## Results

Thirty patients were included. The mean age was 72.4 years and 21 of them were male. Demographic data are summarised in Table 3. All patients presented with Rutherford classification of more than 3 (100%). The median modified PACCS score was 4c. Location of the lesions were superficial femoral, common iliac, popliteal and infrapopliteal arteries. Procedural length varied from 60 to 356 min with a variable fluoroscopy time of 6–93 min (mean 23.44 mins). Follow up ranged between 2 and 28 weeks with 4 patients lost to follow up and 1 patient who died before their outpatient appointment (unrelated to endovascular procedure). Technical success was achieved in 29 out of 30 cases (97%) with re-entry being unsuccessful in one case. A variety of puncture techniques were utilised including; 13 anterograde femoral, 10 retrograde femoral and 7 retrograde popliteal approaches. Some of these procedures required additional punctures namely 1 retrograde, popliteal, one retrograde anterior tibial and 1 antegrade left common femoral artery (CFA). One case had an intraprocedural extravasation which was corrected at the time without further complication. There were no further procedure-related complications. Amputation free survival at 30 days was 97% with one patient having an amputation of their right foot.

**Table 3:** Demographic data of patient cohort

Demographics (*n* = 30)	
Sex	
Male, n (%)	21 (70)
Female, n (%)	9 (30)
Age, mean	72.4
Race	
Caucasian	16
South East Asian	6
Black	2
Other	3
Not stated	3
Risk Factors	
Smoking, n (%)	11 (37)
MI history, n (%)	4 (13)
Diabetes, n (%)	13 (43)
Hypertension, n (%)	20 (67)
Dyslipidaemia, n (%)	17 (57)
Indication	
Claudication, n (%)	9 (30)
Rest Pain, n (%)	8 (27)
CLI, n (%)	2 (7)
Ulcers, n (%)	13 (43)

**Fig. 1:**
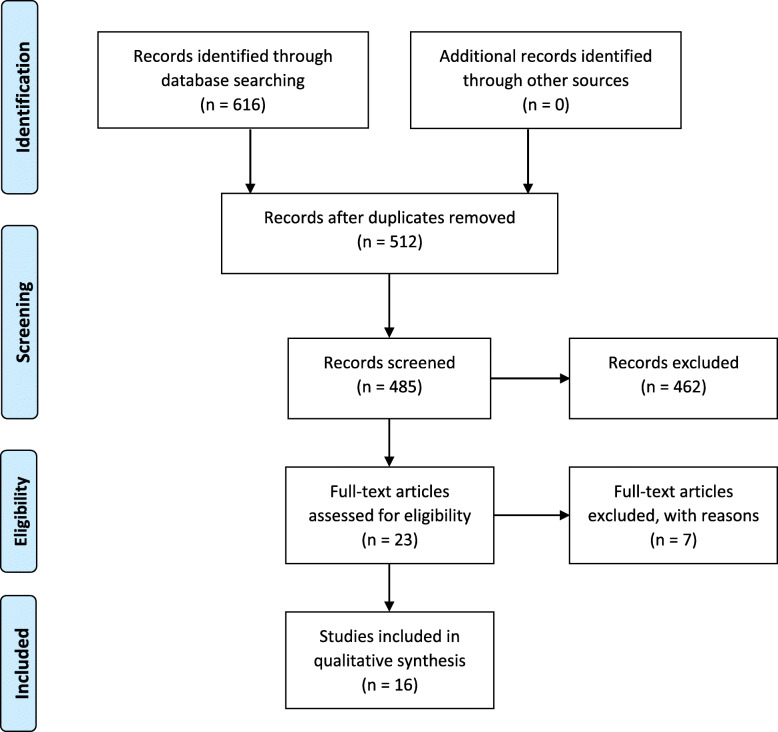
Flowchart of selection process

The search identified 616 studies, of which 593 were excluded due to either duplication, title screening or abstract not meeting the inclusion criteria. The remaining 23 articles were read in full and 7 more articles were excluded for not meeting the inclusion criteria (Fig. 1).

Pioneer-assisted percutaneous recanalization was reported in 430 cases, identified from 16 studies. The reported technical success rate was 87.4–100% and complication rate 0–25.8% (median = 0%). However, due to heterogeneity in definitions of technical success, data could not be pooled for further analysis.

Ten out of the 16 studies reported the length of occlusion with a mean length of occlusion of 14.0 cm for 194 occlusions. Reporting of follow-up was varied, ranging from no mention of follow-up to a maximum of 12 months.

The majority of studies did not define technical success; two studies defined it as 30% or less residual stenosis (Baker et al., 2015; Al-Ameri et al., 2009)and one as 25% or less (Scheinert et al., 2005). Others, defined success as reentry of ≤1 cm from the optimal angiographically defined target vessel (Krishnamurthy et al., 2010) while Vurushkan and colleagues reported an average re-entry distance of 1.75 ± 0.17 cm (Vuruskan & Saracoglu, 2017).

Pioneer was used in various vascular territories. The most commonly reported vessels were the iliac artery (12 studies), followed by the superficial femoral artery (10 studies). It was also successfully used for occlusion of femoropopliteal and infrapopliteal arteries.

Device related complications were noted in in 17 out of the 430 cases (3.9%). Complications reported included manifestation of bleeding at the site of recanalization and angioplasty in four patients at the time of procedure (Jacobs et al., 2006). Vurushkan and colleagues reported complications including two distal embolisations, one external iliac artery perforation and two reinterventions due to acute vessel occlusion whereas Kokkinidis and colleagues reported two dissections (Kokkinidis et al., 2020). Other studies did not describe the nature of the complications reported (Baker et al., 2015; Scheinert et al., 2005).

## Discussion

Collectively, both the data collected from our centre and the data available from the systematic review indicated that the Pioneer Re-entry device is effective as shown by the high success rates and minimal complications.

Randomised control trials comparing treatments for critical limb ischaemia (CLI) are limited, particularly with respect to subinitimal entry and generally compare bypass to angioplasty. BEST-CLI (Farber et al., 2019), a randomised controlled trial does not mandate for percutaneous transluminal angioplasty but allows for the best available endovascular approach to be performed (Farber et al., 2019). As a result, evaluation of specific devices would be difficult. Currently, TASC II guidelines (Zambrano et al., 2014) suggest endovascular recanalization is the standard treatment for total chronic aortoiliac, infrainguinal and infrapopliteal occlusions due to its minimal-invasiveness (Norgren et al., 2007). Kim et al. demonstrated that technical success rates are higher for endovascular treatments such as subintimal treatment than transluminal angioplasty (Kim et al., 2018).

Successful subintimal angioplasty was first described by Bolia and colleagues for femoropopliteal occlusions in 1990 (Bolia et al., 1990). Despite the increasing use of subintimal angioplasty, to the best of our knowledge there are no guidelines or randomised control studies on the recently developed devices such as Pioneer and Outback that are thought to further increase technical success of subintimal angioplasty.

Successful recanalization is of utmost importance in order to achieve good antegrade flow and avoid compromise of collaterals and adjacent vessels, however, reliable assessment of the Pioneer re-entry device based on the existing studies was challenging due to the lack of a standard definition for technical success as well as heterogeneity of reporting other outcomes in the selected papers. The variety in the definition of successful re-entry and the majority of the studies not defining technical success, prevented pooling and statistical analysis of data.

Jacobs and colleagues (Jacobs et al., 2006), the first study to report on the Pioneer device specifically, defined successful re-entry as that of less than 2 cm of the optimal target vessel, demonstrated via angiography (Jacobs et al., 2006). A randomised control study by Gandini et al. 2013, comparing a different re-entry device, Outback, with manual re-entry, defined technical success as re-entry less than 5cm (Gandini et al., 2013) whilst other studies using Pioneer have defined it as less than 1cm (Krishnamurthy et al., 2010). Alternatively, some studies define technical success based on residual stenosis. This poses difficulty in assessing the efficacy and advantage of using a re-entry device over manual re-entry.

We suggest that future research should follow a uniform definition of technical success as re-entry of < 1 cm from the target point, to allow pooling and qualitative analysis of data. The choice of target re-entry distance of < 1 cm is based on the intention to avoid occluding collaterals and/or compromising branches if the dissection extends distal to them which usually presents one of the main limitations of subintimal angioplasty. This is especially important in recanalization of aortoiliac arteries where the inferior mesenteric or renal arteries or potentially, the origin of the lumbar and/or spinal feeding vessels may be compromised (Kitrou et al., 2015). This principle can also be applied for retrograde popliteal approach for long SFA occlusion where compromising the origin of profunda femoris would be of some concern. Our study supports this assertion and found that by making the desired landing zone < 1 cm from the target point, we had no occlusion or compromise of important collateral vessels. As aformentioned we were able to achieve the desired landing zone in 97% of cases (29/30).

The Pioneer catheter has been used in a significant number of studies with a very high success rate and minimal complications. Although the present study had a limited number of cases, it has demonstrated a technical success rate of 97% with 1 minor complication. As stated in previous studies, Pioneer is typically used in cases where manual entry fails. In fact, all the patients of our study had occlusions of high complexity. Moreover, use of IVUS potentially benefits from requiring the expertise of the interventional radiologist. Occlusion chronicity, heavy calcification and length of occlusion are the major contributors to the complexity of the recanalization, yet Pioneer has demonstrated technical success rates of 93% and 100% in occlusions of 23.8 cm and 24.8 cm mean length respectively (Zambrano et al., 2014; Smith et al., 2011). Indeed, the majority of occlusion in our study were over 15 cm (Table 4) and were successfully crossed, therefore, the Pioneer device helps overcome numerous challenges of recanalization.

**Table 4:** Length of occlusion

Length of vessel occlusion	No of patients
**> 5 cm**	**1**
**5–15 cm**	**12**
**> 15 cm**	**17**

Comparing re-entry devices (Pioneer and Outback) with standard crossing techniques; a retrospective study assessing long-term outcomes of common iliac arteries recanalization demonstrated similar target lesion revascularisation, major adverse limb events rates as well as complication rates. In this aforementioned study, Pioneer was used more often than Outback as it is less technically challenging (Kokkinidis et al., 2018). However, we cannot confidently conclude the superiority of one method, as there are no randomised control studies or prospective data collection, comparing the use of Pioneer with using crossing wires alone. Similarly, there are no randomised control studies comparing Outback and Pioneer to assess the differences between the two reentry devices.

The main limitation of Pioneer is the limited availability of IVUS stations in angiography suites. Vessel calcification has been identified as a major cause for RED failure, especially for the Pioneer device as it can reduce ultrasound quality. However, recent studies do not associate calcification with lower success rates for Pioneer (Jacobs et al., 2006; Kokkinidis et al., 2018). The vessel calcification for the patients in this study was calculated using the modified peripheral arterial calcium scoring system (PACSS) which is based on angiographic images (table and reference). The median calcification grade was 4, suggesting supporting the assertion that the Pioneer re-entry system works well despite dense, mixed type calcification.

A factor of success with Pioneer mentioned in only one of the studies is operator familiarity. In the study by Sheikh et al. (Sheikh et al., 2021), it was found that the operators who had used the Pioneer device over 25 times had a 95.8% success rate which dropped to 65.6% in those who had used it 5–25 times. In our experience, 10 cases is probably a reasonable number to gain confidence in the device and technique.

### Limitations of the study

This is a retrospective study and as such there was no standardised follow up. Whilst the fluoroscopy times were available, the procedural time pertinent to the use of the Pioneer device use was not recorded, thus preventing assessment of its impact on operational time. Moreover, the lack of a control group does not allow comparison of technical and clinical success rate between Pioneer and conventional crossing-wire techniques. However, the overall procedure time was not flagged as significantly different from other recanalization procedures in our department.

The clinical follow up time was also largely variable but it must be noted that half of these patients were seen in 2020–2021 during the COVID-19 pandemic where clinic attendance rates both in person and virtually were reduced.

Limitations of the systematic review reflect the heterogeneity and small number of the existing studies. First, all of the studies were retrospective and thus should be interpreted in the context of observational research. Second, statistical analysis and pooling was not possible due to variable reporting outcomes. The study benefited from following adherence to systematic review and PRISMA and including all available data, even those presented in conference abstracts. However, some studies lacked adequate demographic data, definition of success or description of the complications. In addition, the procedural success of re-entry devices such as Outback was grouped together with Pioneer in two studies, therefore preventing extracting pertinent data to Pioneer. The two studies were by Kokkinidis et al., where the Pioneer device was used in 23 cases and outback in 15 cases (and could explain the relatively lower success rate of 90%), as well as Arslan et al., which did not specify which of the seven cases were with the Pioneer device.

## Conclusion

The Pioneer device is safe and effective tool to treat complex arterial occlusions with a high technical success rate and accuracy. However, the cost effectiveness and the exact role of Pioneer and other re-entry devices in total vessel occlusions should be further studied in future prospective trials and or registries using improved and standardised data collection.

## Data Availability

The datasets used and/or analysed during the current study are available from the corresponding author on reasonable request.
